# Arsenic and Other Metals’ Presence in Biomarkers of Cambodians in Arsenic Contaminated Areas

**DOI:** 10.3390/ijerph121114285

**Published:** 2015-11-10

**Authors:** Penradee Chanpiwat, Seiichiro Himeno, Suthipong Sthiannopkao

**Affiliations:** 1 Environmental Research Institute, Chulalongkorn University, Phayathai Road, Pathumwan, Bangkok 10330, Thailand; E-Mail: pchanpiwat@gmail.com; 2 Center of Excellence on Hazardous Substance Management (HSM), Chulalongkorn University, Bangkok 10330, Thailand; 3 Department of Molecular Nutrition and Toxicology, Faculty of Pharmaceutical Sciences, Tokushima Bunri University, Tokushima 770-8514, Japan; E-Mail: himenos@ph.bunri-u.ac.jp; 4 Department of Environmental Engineering, College of Engineering, Dong-A University, 37 Nakdong-Daero 550 Beon-gil, Saha-gu, Busan 604-714, Korea

**Keywords:** arsenic, hair, nails, urine, biomarker, chronic exposure

## Abstract

Chemical analyses of metal (Cr, Mn, Fe, Co, Ni, Cu, Zn, As, Mo, Ba, and Pb) concentrations in hair, nails, and urine of Cambodians in arsenic-contaminated areas who consumed groundwater daily showed elevated levels in these biomarkers for most metals of toxicological interest. The levels of metals in biomarkers corresponded to their levels in groundwater, especially for As, whose concentrations exceeded the WHO guidelines for drinking water. About 75.6% of hair samples from the population in this study contained As levels higher than the normal level in unexposed individuals (1 mg·kg^−1^). Most of the population (83.3%) showed As urinary levels exceeding the normal (<50 ng·mg^−1^). These results indicate the possibility of arsenicosis symptoms in residents of the areas studied. Among the three biomarkers tested, hair has shown to be a reliable indicator of metal exposures. The levels of As (*r^2^* = 0.633), Ba (*r^2^* = 0.646), Fe (*r^2^* = 0.595), and Mo (*r^2^* = 0.555) in hair were strongly positively associated with the levels of those metals in groundwater. In addition, significant weak correlations (*p* < 0.01) were found between levels of exposure to As and As concentrations in both nails (*r^2^* = 0.544) and urine (*r^2^* = 0.243).

## 1. Introduction

In complex modern societies, humans can be exposed on a daily basis to a wide range of chemicals and pollutants. These come not only from environmental sources such as air, soil, water, and food, but also from a variety of lifestyle and occupational activities [1]. Exposure to environmental pollution can be considered a main source of adverse human health impacts, around the globe. These negative health impacts are generally higher in the developing countries, the poorer ones more so, as poverty’s consequences tend to include lack of investment in adequate treatment technology and weak environmental law enforcement [1]. Exposure to toxic chemicals in groundwater, for example arsenic and fluoride in East and Southeast Asian countries, is a well-known manifestation of this situation [2].

Since 1999, numerous incidents have been reported of groundwater contamination in Cambodia by metals such as arsenic (As), manganese (Mn), and barium (Ba), occurring in provinces located along the Mekong delta floodplains [3,4,5,6,7,8,9]. As the groundwater constitutes a significant drinking water source for Cambodians in rural areas (some 53%), approximately 100,000 are believed exposed daily to several toxic metals through the pathway of ingestion [8,9]. Consumption of contaminated food such as rice and fish also contributes to metal exposure via the ingestion pathway [1]. Chronic exposure to such metals, even at low levels, can lead to disabilities, diseases, or even death [1,10,11].

One way to assess the extent of environmental exposure to chemicals, and to screen their potential impacts on humans, is by conducting human biomonitoring (HBM). HBM has been a tool of interest for quantifying human exposure, and body responses, to environmental pollutants from as far back as the 19th century [12,13,14,15,16]. Numerous studies have employed HBM to investigate As exposure through drinking water. These have reported significant relationships between levels of As exposure and levels of As determined in several biomarkers, including in blood, urine, skin-scales, hair, and nails [17,18,19,20,21]. Still, research on the agreement of As concentrations found among the different biomarkers and the inter-relationships between As and other elements within each biomarker is still very limited. The objectives of this study were therefore to (i) determine the levels and relationships of metals in the hair, nails, and urine of Cambodia residents consuming As enriched groundwater; (ii) study the inter-relationships between As and other elements in each type of biomarker; and (iii) to confirm the agreement of As environmental exposure levels and As concentrations in different biomarkers.

## 2. Materials and Methods

### 2.1. Study Areas

**Figure 1:**
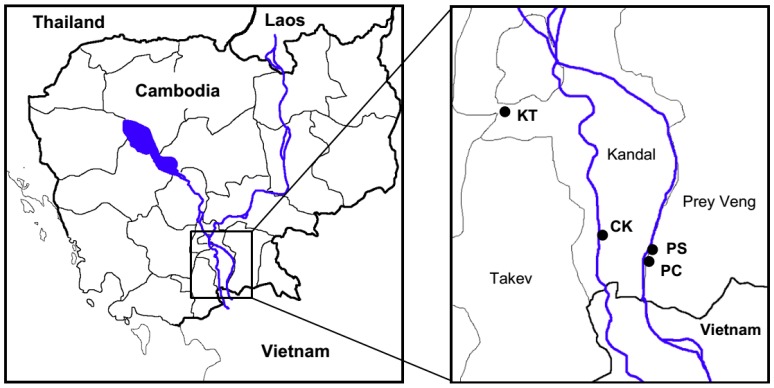
Locations of all areas studied along the Mekong River.

Four villages located in two provinces in the southern part of Cambodia were selected for this present study (Figure 1). Based on a preliminary survey of As concentrations in groundwater conducted by a local Non-Governmental Organization, the villages Sambour (PS) and Preak Chrov (PC) in Prey Veng Province, as well as Chang Kaoh (CK) in Kandal Province, where As concentrations exceeded the WHO drinking water quality guideline of 10 μg·L^−1^, were selected as As contaminated areas. The village of Kampong Toul (KT) in Kandal Province was selected as a non-polluted (control) area. 

### 2.2. Collection of Human Biomarkers

The ethical approval for conducting health risk assessment using hair, nails, and urine of Cambodia residents in all areas studied was approved by the National Ethics Committee for Health Research (NECHR), Ministry of Health, Cambodia (Reference No. 180 NECHR). 

A total of 180 individuals (50 from each contaminated area and 30 from the control area) were recruited as study subjects. The subject inclusion criteria used in this present study were a period of habitation in the study areas, as well as a period of continuous groundwater consumption, of at least one year. Prior to collection of biomarkers, each subject was informed by a local researcher about the purpose of this study with a document written in Khmer. In addition, the informed consent of each subject was obtained before the collection of hair, nails, and urine samples. Hair samples of about 1 cm were collected from the region of the head close to the scalp behind the ear. Nail samples were collected primarily from toenails. In very rare cases, (one case from CK and three cases from KT) samples of other nails supplemented these when the amount of toenail sample was not enough. For the urine samples, spot urine was collected from each subject and immediately kept on ice. After that, all urine samples were transferred to a liquid nitrogen tank and delivered to a laboratory.

### 2.3. Collection of Groundwater

At the same time, as the biomarker collection, 76 groundwater samples were collected from tube-wells used as a source of drinking water by subjects in the study. All groundwater samples were collected after 5 min of pumping. Once collected, each sample was filtered, acidified with nitric acid to a pH of less than 2, then stored in an acid-washed tube until analysis.

### 2.4. Sample Pre-treatment 

In the laboratory, hair and nail samples were first cleaned according to the method described by Chen *et al.* (1999) [22]. In brief, hair and nail samples were immersed in 1% Triton X-100, sonicated for 20 min, washed 5 times with milli-Q water, and finally dried at 60 °C overnight in a drying oven. After that, all cleaned hair and nail samples were digested for total metal concentration with concentrated nitric acid (electrophoresis (EL) grade, an acid with very stringently limited concentrations of metallic impurities, purchased from Kanto Chemical Co, Inc., Tokyo, Japan) using a hot plate digestion method at 70 °C for 15 min and 115 °C for 15 min [23]. The final washing solution was collected to ensure that there were no particles or dust attached to cleansed hair. Concentrations of all metals of interest in the final washing solution were lower than the limit of detection for inductively coupled plasma mass spectrometry (ICP-MS; HP-7700x, Agilent Technologies, Kanagawa, Japan). For spot urine samples, all samples were digested in the same manner as the hair and nail sample digestion. After digestion, samples were diluted with 1.0% nitric acid and analyzed by ICP-MS. 

In addition, a certified reference material (CRM) of cod fish tissue (National Metrology Institute of Japan (NMIJ) CRM 7402-a, No. 20) was also digested and analyzed by ICP-MS in the same manner as all biomarker samples to ensure the effectiveness of metal recovery by the digestion method. The percentage recoveries of all metals of interest were in the range of 83.6% to 86.9%. Procedural blanks were also treated and analyzed in the same manner as all samples pre-treatment. The accuracy of instrument analysis was conducted by analyzing the standard reference material (SRM) of trace elements in water (SRM 1643e) purchased from National Institute of Standards and Technology (NIST), USA and analytical blanks after the analysis of every 20 samples. The percentage recoveries of instrumental analyses obtained were in the range of 85.7% to 105.1%. Levels of all metals in procedural and analytical blanks were found to be lower than the limits of quantification as reported in Table 1.

**Table 1:** Limits of quantification (LOQ) for metals in groundwater and biomarkers.

Element	Limit of Detection		
	Groundwater (μg·L^−1^)	Hair and Nails (mg·kg^−1^)	Urine (ng·mg^−1^ Creatinine)
Cr	0.38	0.10	0.10
Mn	1.20	0.33	0.07
Fe	2.78	2.93	0.09
Co	0.32	0.01	0.04
Ni	0.34	0.02	0.01
Cu	0.29	0.12	0.04
Zn	0.33	0.14	0.09
As	0.55	0.03	0.02
Mo	0.35	0.02	0.09
Ba	1.36	0.79	0.02
Pb	0.33	0.01	0.02

### 2.5. Sample Analyses

Concentrations of metals contained in all water samples and in digested biomarker (hair, nail, and urine) samples were determined by ICP-MS with an online addition of germanium (1 ppm) as an internal standard. The ion signal for germanium at m/z 72 was found. The limits of quantification for metals in groundwater, hair, nail, and urine samples are summarized in Table 1. Duplications of all sample digestions and analyses were employed in this study.

Metal concentrations in urine were also determined by ICP-MS. Afterward their metabolized concentrations were adjusted with urinary creatinine and measured by a specific gravity method, to finally obtain the metal levels in urine in units of ng·mg^−1^ creatinine.

### 2.6. Data Analyses

All statistical analyses were performed using the Statistical Packages for Social Sciences (IBM SPSS) Software (version 15.0). Prior to statistical analysis, all data sets were tested for normality of distribution by the Komogorov-Smirnov test (*n* > 50). Since the distributions of the data sets were not normal, a nonparametric test, the Kruskal-Wallis H, was applied to assess the regional differences in metal concentrations in groundwater, hair, nails, and urine. The correlation between each metal’s concentration in groundwater and its corresponding concentration in a given biomarker, as well as the correlation of concentrations across biomarkers for a given metal, were analyzed by the Spearman’s rho correlation statistic. The value of *p* < 0.01 was used to judge the significance of differences and correlations for the parameters mentioned.

## 3. Results and Discussion

### 3.1. Metals Concentrations in Groundwater

Dissolved concentrations of metals in groundwater are summarized in Supplementary Materials (Tables S1–S4). 

Overall, significant differences could not be found among concentrations of all metals except Fe, As, and Mo in groundwater collected from all areas studied. Compared to the control area (KT), significantly higher concentrations of Fe, As, and Mo were found in samples collected from contaminated areas. This finding confirmed that the mobilization of As in study areas, floodplains along the Mekong River, was mainly caused by the desorption of As from As-bearing Fe oxides in sediments [8]. For this reason, high levels of Fe and As were determined in groundwater samples collected from all contaminated areas in the present study. Of the 76 collected groundwater samples, 84.2% exceeded WHO (World Health Organization) drinking water guidelines for As concentrations, and 35.9% exceeded the guidelines for Ba. Since the WHO currently has regulations only for As (10 μg·L^−1^) and Ba (700 μg·L^−1^) concentrations in groundwater [24], only percentages of groundwater samples containing As and Ba higher than the WHO guidelines were reported. All groundwater samples collected from PS, PC, and CK villages were found to be heavily contaminated by As. It was found that 100% of samples collected from CK, PC, and PS, the contaminated areas, contained As higher than the WHO guideline of 10 μg·L^−1^. Ranges of concentrations of As in groundwater from the villages were 16.02–959 μg·L^−1^ for PS, 57.9–997 μg·L^−1^ for PC, and 402–923 μg·L^−1^ for CK. (Tables S2–S4). For Ba, the percentages of samples collected from CK and PC which were contaminated with concentrations higher than the WHO guideline were 37.5% and 63.6%, respectively. All samples collected from the control area, KT, showed levels of As and Ba well below the WHO guidelines. Concentrations of As determined in groundwater collected from the contaminated villages (PS, PC, and CK) in this study were of the same magnitude as those found in previous research conducted in both Prey Veng and Kandal provinces. For Prey Veng Province, it should be noted that As concentrations found in this study were about 2.8 times higher than the most recently reported As concentrations contained in groundwater collected from that province’s Svay Chrum village (in the Peam Chor district) [25]. In Kandal Province, well water samples in the Kien Svay district and in Koh Thom contained As in concentrations reaching up to 943 μg·L^−1^ and 1,832 μg·L^−1^, respectively [21,26]. Phan *et al.* (2013) [26] also reported high Ba concentrations (872–2653 μg·L^−1^) in groundwater collected from As contaminated areas. The levels of Ba in groundwater found in the present study were similar to those reported earlier (24.55–2631 μg·L^−1^).

### 3.2. Metal Concentrations in Hair 

The median concentrations of metals in all collected hair samples were 0.29 mg·kg^−1^ for Cr, 17.92 mg·kg^−1^ for Mn, 79.98 mg·kg^−1^ for Fe, 0.06 mg·kg^−1^ for Co, 0.70 mg·kg^−1^ for Ni, 12.52 mg·kg^−1^ for Cu, 255.4 mg·kg^−1^ for Zn, 4.18 mg·kg^−1^ for As, 0.07 mg·kg^−1^ for Mo, 10.39 mg·kg^−1^ for Ba, and 2.64 mg·kg^−1^ for Pb. The orders of metal concentrations found in hair were Co < Mo < Cr < Ni < Pb < As < Ba < Cu < Mn < Fe < Zn. Statistical analyses revealed significant differences in concentrations of all metals in hair samples collected from the areas studied. Only two areas (CK and PC) were identified with metals concentrations in hair which were statistically higher than the other areas. Hair samples collected from PC were found with Mn, Fe, Cu, As, Ba, and Pb higher than in the samples collected from the other three areas (KT, CK, and PS) (Tables S1–S4). The high levels of these metals in hair corresponded to the high levels of these same metals found in groundwater samples collected from the PC area. Groundwater As concentrations in the CK area were higher than those in PC. However, higher significant differences were not obtained (*p* > 0.01). Along with the hair samples from KT, the groundwater samples from KT also yielded the lowest levels of Fe, As, Mo, and Ba among the four areas. It has been observed that the level of metals in groundwater is not the only factor affecting the accumulation of metals in biomarkers; other important factors include age, gender, individual susceptibility, period of groundwater consumption and amount of daily groundwater consumption [18,20].

Generally, As is the element whose concentration is most often observed in hair and other types of biomarkers. Because earlier studies did not include levels of metals other than As in hair, only the hair As concentrations in this study could be compared to previous researchers’ findings. 

The ATSDR (Agency for Toxic Substances and Disease Registry) recommends the normal level of As in hair of unexposed individuals should be less than 1 mg·kg^−1^ [27]. This As level has accordingly been a convenient, continuously employable indication of the threshold level of arsenicosis symptoms. In the present study, 91.3% of all subjects living in the As contaminated areas had hair As higher than this threshold level. No one living in the non-contaminated (KT) area was found with a hair As concentration higher than 1 mg·kg^−1^. The percentage of subjects in the non-control areas measuring As in hair higher than the 1 mg·kg^−1^ level was 90% in CK, 94% in PC, and 88% in PS. These figures indicate the possibility of As toxicity among residents of the areas who chronically consume groundwater. The range of As concentrations in hair in this study (0.03 to 56.75 mg·kg^−1^) was comparable to those reported for Cambodian subjects’ hair in previous studies. For example, the range of As concentrations in hair collected from the residents of As contaminated areas had been previously measured at 0.01–57.21 mg·kg^−1^ [8], 0.10–7.95 mg·kg^−1^ [21], and 0.06–30 mg·kg^−1^ [28]. Sampson *et al.* (2008) [29] found hair As concentrations in a Cambodian village where arsenicosis symptoms had been diagnosed to be about 2.1–13.94 mg·kg^−1^. The ranges of As in hair in CK, PC, and PS were respectively 0.65–56.75 mg·kg^−1^_,_ 0.27–22.69 mg·kg^−1^_,_ and 0.23–12.94 mg·kg^−1^, (Table 2). The three non-control village areas investigated in this present study therefore seem vulnerable to toxicity from chronic arsenic exposure. 

**Table 2:** As concentrations in groundwater and biomarkers in four provinces of Cambodia.

Statistics	Population	Study Area			
		KT	CK	PC	PS
**Groundwater** (μg·L^−1^)					
No. of samples	76	12	24	22	18
Min	<LOQ	<LOQ	402	57.93	16.02
Max	997	8.36	923	997	959
Mean	512	2.56	686	587	567
Median	591	1.24	700	650	616
SE	22.13	0.44	19.23	32.68	32.04
**Hair** (mg·kg^−1^)					
No. of samples	180	30	50	50	50
Min	0.03	0.03	0.65	0.27	0.23
Max	56.75	0.41	56.75	22.69	12.94
Mean	5.93	0.17	9.69	7.37	4.19
Median	4.18	0.16	6.37	6.78	4.12
SE	0.52	0.02	1.44	0.79	0.36
**Nails** (mg·kg^−1^)					
No. of samples	176	27	49	50	50
Min	0.11	0.11	0.77	1.14	0.55
Max	23.24	0.88	23.24	18.48	14.41
Mean	4.14	0.37	5.47	4.72	4.29
Median	3.04	0.31	3.84	3.40	3.61
SE	0.30	0.04	0.72	0.49	0.45
**Urine** (ng·mg^−1^ creatinine)					
No. of samples	180	30	50	50	5
Min	22.26	30.22	22.26	52.51	46.76
Max	995	995	448	689	407
Mean	124	107	81.07	199	103
Median	86.77	60.04	64.40	159	84.02
SE	9.21	32.32	8.64	20.63	8.97

SE = Standard error of the mean.

**Figure 2:**
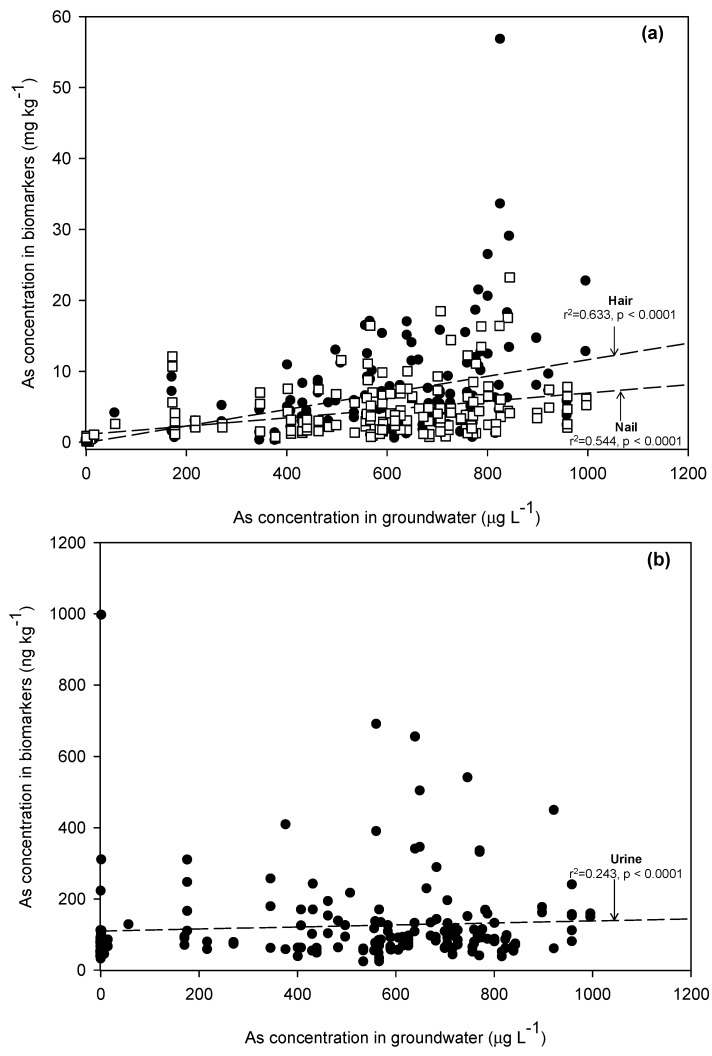
(**a**) Relationships of As in groundwater to biomarkers in hair and nails. (**b**) Relationships of As in groundwater to biomarkers in urine.

**Figure 3:**
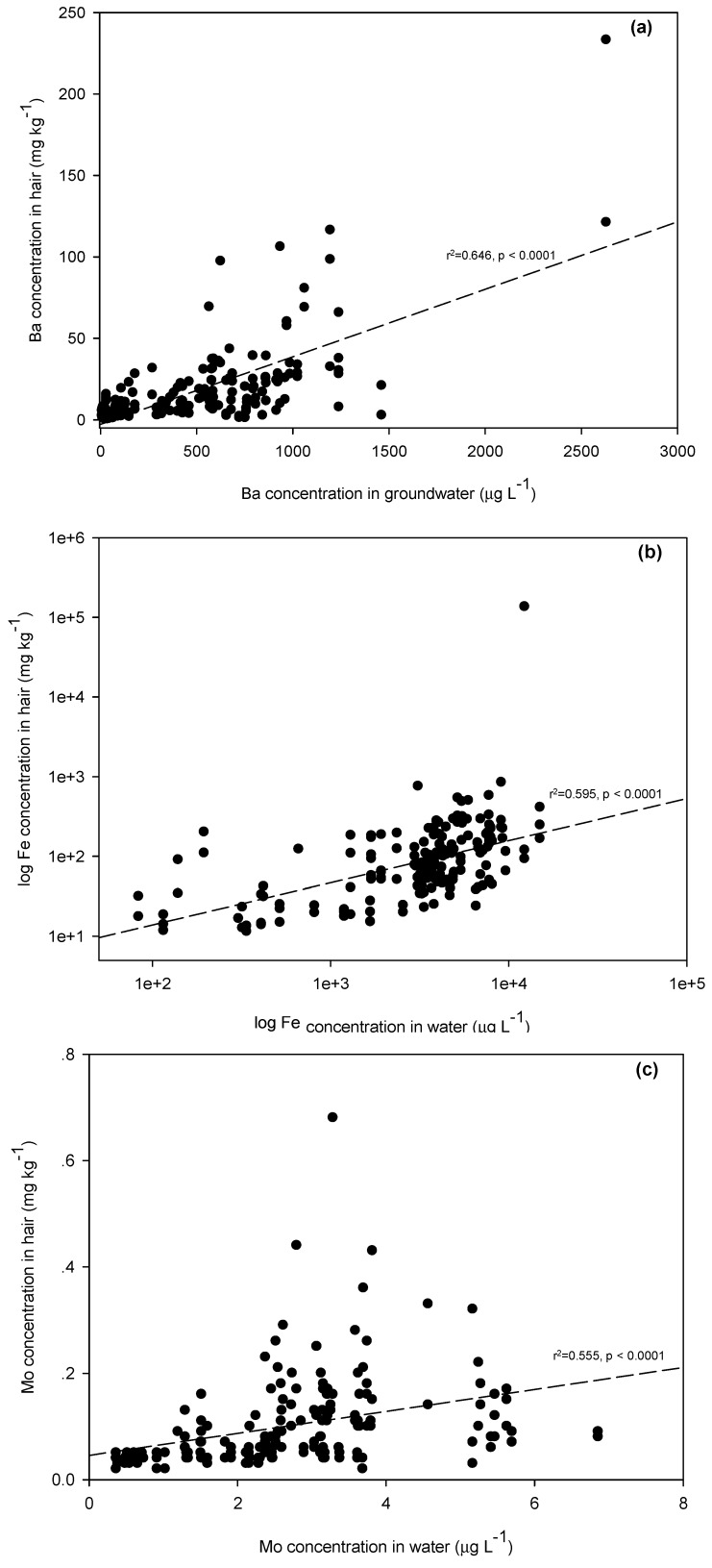
Relationships of (**a**) Ba, (**b**) Fe, and (**c**) Mo in groundwater to hair samples.

In addition, Table 3 summarizes the significant correlations between levels of As, Ba, Cr, Mn, Fe, Ni, and Mo in groundwater and the levels of those same metals present in the hair samples of subjects who consumed groundwater on a daily basis. The levels of As (*r^2^* = 0.633) (Figure 2a), Ba (*r^2^* = 0.646) (Figure 3a), Fe (*r^2^* = 0.595) (Figure 3b), and Mo (*r^2^* = 0.555) (Figure 3c) in hair were moderately positively associated with the levels of these metals in the groundwater, which all residents in this study relied on as their sole source of drinking water. It is also worth noting that the highest Fe concentration (134,700 mg·kg^−1^) in hair was in a male resident who had been exposed to the highest level of Fe in groundwater (12,350 μg·L^−1^). The results clearly indicated that groundwater used as drinking water was the source of various metal exposures. Since the desorption of As from As-bearing Fe (-Mn) oxides in sediments was the key factor mobilizing As in groundwater, the higher exposure and accumulation in biomarkers was observed. 

### 3.3. Metal Concentrations in Nails

The rank ordering of metal concentrations found in nails was somewhat similar to that for hair. The median levels of metals in nails, from lowest to highest concentrations, were 0.05 mg·kg^−1^ Mo, 0.35 mg·kg^−1^ Co, 1.30 mg·kg^−1^ Pb, 1.40 mg·kg^−1^ Ni, 1.64 mg·kg^−1^ Cr, 3.04 mg·kg^−1^ As, 4.95 mg·kg^−1^ Cu, 10.07 mg·kg^−1^ Ba, 25.46 mg·kg^−1^ Mn, 113.0 mg·kg^−1^ Zn, and 875.7 mg·kg^−1^ Fe. Concentrations of Cr, Mn, Fe, Co, Ni, Cu, and Ba in nails collected from the PC area were significantly higher than in those from the other study areas. This is in agreement with the metal concentrations measured in groundwater samples, where the higher concentrations were usually detected in the PC area (Table 2, Table S3). Significantly lower Cr, Mn, Fe, Co, Ni, Cu, Zn, As, Ba, and Pb concentrations were found in samples collected from the control area. 

For As concentrations in nails, the ATSDR also recommends 1 mg·kg^−1^ as the normal level for unexposed individuals [27]. In this study, only nail samples collected from the uncontaminated control area (KT) contained As levels lower than this normal level (Table 2). About 96.0% of the entire subject population living in the As contaminated areas had As levels in nails higher than the ASTDR recommended level. Overall, most concentrations of As in nails found in this present study did fall within the range of background As levels in nails (<1.5 to 7.7 mg·kg^−1^) as noted by Hughes (2006) [30]. However, 10.7% of the population in this study was found with As levels in nails higher than the background range, among which the upper end of As concentrations was about 3 times higher than this background level. 

In addition, significant relationships between As, Ba, Fe, and Ni concentrations in nail and groundwater samples were determined (Table 3). However, a strong positive correlation was found only for As (*r^2^* = 0.544) (Figure 2a).

### 3.4. Arsenic Concentrations in Urine

ICP-MS analyses of metal concentrations in urine revealed different orderings compared to those of both hair and nail samples. The levels of metals found in urine, ranged from lowest to highest, were 0.36 ng·mg^−1^ Co, 1.23 ng·mg^−1^ Mn, 1.69 ng·mg^−1^ Cr, 2.38 ng·mg^−1^ Pb, 8.85 ng·mg^−1^ Ni, 11.10 ng·mg^−1^ Cu, 12.33 ng·mg^−1^ Ba, 38.63 ng·mg^−1^ Fe, 65.87 ng·mg^−1^ Mo, 86.77 ng·mg^−1^ As, and 388.7 ng·mg^−1^ Zn. However, Mn, Fe, Ni, and As concentrations were found to be statistically different among the study areas (Tables S1–S4). No good agreement between metal concentrations in groundwater and in urine was found. However, lower metal concentrations in individuals’ urine were reported in the urine samples collected from uncontaminated areas. Urine samples from PC village, which had the highest As groundwater concentration, were also highest in As (Table S3. It can be concluded that the higher the As exposure level, the higher the excretion of As from the human body, since As has no essential metabolic function. Though the background levels of urinary As can be found within the range of 5 to 50 ng·mg^−1^,excessive exposure to As in groundwater could lead to urinary As levels exceeding 700 ng·mg^−1^ [30]. In the control KT area, 57.1% of urinary samples had As higher than the background level, as did 65.2% in CK, 100% in PC, and 100% in PS. Furthermore, about 1.2% of the whole population in this study was found with urinary As higher than 700 ng·mg^−1^. An epidemiological study of environmental As exposure in Kandal Province has shown that the urinary As levels in both asymptomatic (73.04 ± 52.24 ng·mg^−1^) and arsenicosis patients (78.74 ± 69.84 ng·mg^−1^) were not significantly different [18]. Noting that the levels of urinary As from subjects in KT village consuming As-free groundwater (0.54–8.36 μg·L^−1^) were not statistically different from those of subjects consuming As-enriched groundwater, the results of urinary As obtained from this present study could not solely be used to indicate the level of As exposure or predict the development of individual As health impacts. Phan *et al.* (2014) [18] concluded that though villagers were not exposed to As contaminated groundwater, they could still be exposed to As, particularly through foodstuffs. Only one weakly positive correlation (*r^2^* = 0.243), which was for As, was found between levels of metal exposure and concentrations of metal in urine (Table 3 and Figure 2b).

### 3.5. Comparison of Metal Concentrations in Biomarkers and Agreement among Different Biomarkers 

Two other studies, by Samanta *et al.*, 2004 [20] and Coelho *et al.*, 2012 [31], have looked at concentrations of other metals in addition to As in biomarkers of individuals who were exposed environmentally and occupationally. Concentrations of most metals of interest in this study were even higher than in those researchers’ populations (in India and Portugal). Groups of Indians had been exposed to Mn, Fe, and Zn through ingestion of groundwater [20]. Concentrations of these same metals in hair and in nail samples in the present study were respectively about 1.8 to 12.4 and 1.2 to 1.6 times higher than in the Indian residents. Taking another comparison, concentrations of Cr, Mn, and Pb contained in hair and nails of Cambodian subjects in this study were about 1.6 to 22.3 times higher than the concentrations of these metals in the same biomarkers in workers in the mining areas of Portugal [31]. These results strongly confirm the relatively high level of metals exposure experienced by our subject population. Individuals exposed to these metals will have accumulated some amounts of them in their tissues, in addition to their presence in biomarkers. 

To compare the different levels of metals contained in different biomarkers within each individual studied, a Wilcoxon Signed-Rank Test was performed. A statistical analyses (by pairwise comparisons) revealed significant differences (*p* < 0.01) in the individual median concentrations of Fe, Co, Ni, Zn, As, and Mo in all three biomarkers. The negative Z-values shown in Table 4 tell that the statistics are based on *negative ranks*, meaning when there was an increase in a specific metal concentration in one biomarker, there was a significant decrease in the same metal concentration in the other biomarker. This finding fits with the notion of extensive biotransformation of ingested As by the human body. Specifically, because the body can excrete about 40% to 70% of the total ingested inorganic As within about 48 h [8], the higher the amount of As excreted the lower the amount of As accumulating in hair and nails. Table 4 also summarizes the varying rank orders of metal concentrations for each pair of biomarkers. Note that for different metals, different sequences of biomarkers would retain the highest to lowest concentrations. Table 5 displays correlations of concentrations of the same metal as found in two biomarkers, reflecting the relative propensity of each to retain the metal following exposure. 

**Table 3:** Correlations of metal concentrations in biomarkers to concentrations in groundwater.

Biomarker	As	Ba	Cr	Mn	Fe	Co	Ni	Cu	Zn	Mo	Pb
Hair	0.633 **	0.646 **	−0.388 **	0.234 **	0.595 **	0.149	−0.287 **	−0.017	0.132	0.555 **	0.027
Nail	0.544 **	0.306 **	−0.111	−0.073	0.296 **	−0.176 **	−0.202 **	−0.076	0.008	0.082	−0.004
Urine	0.243 **	−0.104	0.018	0.008	0.033	0.112	−0.014	−0.103	−0.014	0.122	0.031

** Correlation is significant at the 0.01 level (2-tailed).

**Table 4:** Comparisons of metal concentrations in each biomarker for assessed subjects.

Metal	Statistical Analyses for Comparisons Of Metal Concentrations in Each Pair of Biomarkers (Z− and *p*−Values)			Order of Biomarkers by Concentration of Each Metal
	Hair−Nail	Hair-Urine	Nail-Urine	
Cr	−10.459; <0.0001	−10.596; <0.0001	−2.215; 0.027	Hair < Nail < Urine
Mn	−1.970; 0.049	−10.738; <0.0001	−10.617; <0.0001	Urine < Hair < Nail
Fe	−11.096; <0.0001	−4.868; <0.0001	−11.237; <0.0001	Urine < Hair < Nail
Co	−10.992; <0.0001	−11.557; <0.0001	−1.337; <0.0001	Hair < Nail < Urine
Ni	−6.158; <0.0001	−11.456; <0.0001	−11.078; <0.0001	Hair < Nail < Urine
Cu	−10.565; <0.0001	−2.247; 0.025	−9.728; <0.0001	Urine < Hair < Nail
Zn	−10.954; <0.0001	−3.560; <0.0001	−11.272; <0.0001	Nail < Hair < Urine
As	−3.427; <0.0001	−11.635; <0.0001	−11.505; <0.0001	Nail < Hair < Urine
Mo	−7.434; <0.0001	−11.635; <0.0001	−11.505; <0.0001	Nail < Hair < Urine
Ba	−1.848; 0.065	−0.895; 0.371	−2.649; 0.008	Nail < Hair < Urine
Pb	−7.837; <0.0001	−2.103; 0.035	−6.093; <0.0001	Urine < Hair < Nail

**Table 5:** Correlations of metal concentrations among biomarkers.

Biomarker	As	Ba	Cr	Mn	Fe	Co	Ni	Cu	Zn	Mo	Pb
Hair-Nail	0.721 **	0.341 **	0.067	0.315 **	0.358 **	0.203 **	0.079	0.102	−0.019	0.079	0.276 **
Hair-Urine	0.275 **	−0.069	0.023	0.057	0.099	0.038	−0.034	−0.018	−0.079	0.069	−0.026
Nail-Urine	0.297 **	−0.112	−0.077	−0.096	−0.030	−0.084	0.033	−0.082	0.078	0.006	−0.134

** Correlation is significant at the 0.01 level (2-tailed).

**Figure 4:**
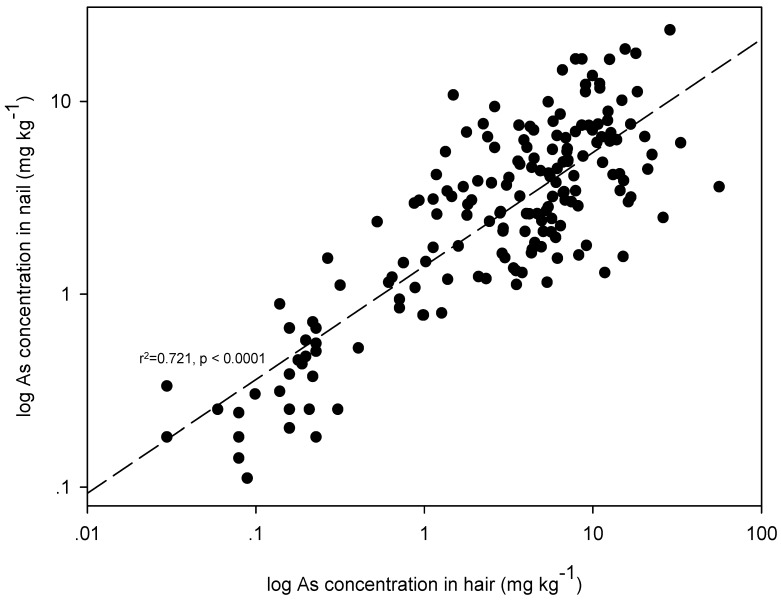
The relationship of As levels in hair and nails.

As shown in Table 5, significant (*p* < 0.01), though weak, correlations between concentrations found in hair and nails occurred for Ba (*r^2^* = 0.341), Mn (*r^2^* = 0.315), Fe (*r^2^* = 0.358), Co (*r^2^* = 0.203), and Pb (*r^2^* = 0.276). Since hair and nails are rich in keratin, which has a high affinity for As, elevated concentrations of As were, as expected, found. Thus, keratin containing cysteine residue with sulfhydryl groups can result in As and sulfur complexation in both hair and nails [20]. Hair and nails in a similar manner incorporate other elements such as Fe, Mn, Ni, and Pb. The results of this study agree well with those of Samanta *et al.* (2004) [20], who found that Zn, Cu, Fe, and Mn, which are associated with enzymes, generally show higher ranges of concentrations in hair, nails, and skin-scale than the other elements do. In addition, there were significant positive correlations between amounts of As in all the different biomarker pairs (Hair-Nail: *r^2^* = 0.721 (Figure 4), Hair-Urine: *r^2^* = 0.275, Nail-Urine: *r^2^* = 0.297). For Cr, Ni, Cu, Zn, and Mo, such strong relationships between amounts in hair, nails, and urine pairs could not be observed. It can be concluded from the results of such correlation analyses that hair can be considered a good biomarker for degree of environmental exposure to a variety of metals. In particular, As levels in hair and in nails can be used to indicate chronic exposure arsenic [32]. Levels of metals in urine, especially As, can be considered to indicate only recent exposure; urinary As is usually eliminated within 3–4 days [33]. The sample collection, treatment, and analysis of hair are moreover easy, practical, ethically innocuous, cost-efficient, and of high toxicological relevance compared, on these attributes, to sampling nails and urine. [15,16]. 

The As concentrations in urine collected from subjects in the non-contaminated area (KT) and those collected in the contaminated areas (CK, PC, and PS) were not statistically different can be explained by the consumption in KT of contaminated food, especially fish and rice cultivated in Kandal, the province where KT village is located. It was reported by Wang *et al.* [34] that the daily intake of arsenic via food consumption in Kandal would contribute approximately 604 μg·day^−1^ of As exposure. Interestingly, fish consumption was found to account for the greatest proportion of total As daily intake in Kandal. Data on individual residents’ fish consumption in the KT area supported this hypothesis well, with subjects in this study ordinarily consuming fish three to seven times weekly.

## 4. Conclusions

This study was conducted in As-contaminated areas of Cambodia, where most groundwater drinking water sources contain As concentrations higher than 10 μg·L^−1^. Elevated levels of As in biomarkers among the local population were therefore expected. Measured concentrations of As in the hair, urine, and nails of subjects in these areas were higher than for a control area or background levels. Other metals, including Fe, Mn, Cr, Ni, and Pb, were also detected at elevated levels in all biomarkers. The levels of metals accumulated in biomarkers corresponded to the levels of those metals in the groundwater of each study area. This study, while dealing with As-enriched groundwater, demonstrates the importance of measuring the concentrations of other elements along with As. 

The levels of As in all biomarkers among exposed subjects indicate medical examinations with diagnostic procedures for arsenicosis should be regularly conducted to allow timely intervention for symptomatic individuals. In addition, the consumption of As-enriched groundwater should be banned and As-free drinking water provided in the affected areas to reduce the level of As exposure. For a better understanding of metals toxicity, how the interactions among these elements may alter their toxicological effects should be further studied.

## Supplementary Files

Supplementary File 1
