# Supplementary material for: Arsenic and Other Metals’ Presence in Biomarkers of Cambodians in Arsenic Contaminated Areas
Source: Int J Environ Res Public Health. 2015 Nov 10;12(11):14285–300. doi: 10.3390/ijerph121114285 (PMC4661647; doi:10.3390/ijerph121114285)
Supplement: Supplementary File 1 [file ijerph-12-14285-s001.pdf]

Arsenic and Other Metals' Presence in Biomarkers of Cambodians in Arsenic Contaminated Areas

Table S1. Metal concentrations in groundwater, hair, fingernails, and urine of KT.

**Statistical Value**	**Total Concentration**										
	**Cr**	**Mn**	**Fe**	**Co**	**Ni**	**Cu**	**Zn**	**As**	**Mo**	**Ba**	**Pb**
**Groundwater ($\mu g \cdot L^{-1}$)**											
Min	<LOQ	8.57	83.91	<LOQ	<LOQ	0.60	1.70	<LOQ	0.37	9.48	<LOQ
Max	20.68	4993	2569	8.64	12.21	9.32	142	8.36	1.34	295	6.43
Mean	2.91	586	703	1.08	1.82	2.04	20.41	2.56	0.67	52.49	1.36
Median	1.23	302	411	0.59	0.95	1.12	6.20	1.24	0.61	30.59	0.38
SE	0.91	220.57	129.72	0.38	0.53	0.40	6.42	0.44	0.05	12.82	0.35
**Hair ($mg \cdot kg^{-1}$)**											
Min	<LOQ	0.68	11.36	0.01	0.12	3.98	96.09	0.03	0.02	0.31	0.29
Max	0.84	92.72	89.95	0.22	1.15	16.38	701	0.41	0.05	15.47	19.67
Mean	0.21	15.76	22.32	0.05	0.31	10.12	202	0.17	0.04	3.31	2.97
Median	0.17	7.99	19.64	0.20	0.24	9.83	180	0.16	0.04	2.33	1.80
SE	0.03	4.27	2.62	0.01	0.01	0.47	19.57	0.02	0.01	0.58	0.68
**Nails ($mg \cdot kg^{-1}$)**											
Min	0.32	0.99	49.15	0.03	0.33	1.27	50.59	0.11	0.02	0.85	0.27
Max	6.28	133	1345	0.61	3.05	26.06	173	0.88	0.11	35.93	16.05
Mean	1.40	13.72	259	0.13	1.18	5.37	115	0.37	0.05	4.47	1.92
Median	0.93	6.77	197	0.10	1.04	4.31	115	0.31	0.04	2.88	0.93
SE	0.24	4.95	52.03	0.02	0.15	0.85	5.65	0.04	0.01	1.29	0.57

Table S1. *Cont.*

Statistical Value	Total Concentration										
	**Cr**	**Mn**	**Fe**	**Co**	**Ni**	**Cu**	**Zn**	**As**	**Mo**	**Ba**	**Pb**
**Urine (ng·mg^{-1} creatinine)**											
Min	0.64	0.25	13.09	0.15	1.98	5.32	97.14	30.22	11.14	2.14	1.56
Max	72.94	216	317	1.84	41.80	53.55	924	995	251	79.65	8.26
Mean	6.14	9.00	70.64	0.50	10.93	12.21	315	107~~.10~~	63.63	23.26	3.94
Median	2.04	1.33	41.44	0.42	8.17	11.04	270	60.04	58.86	19.62	3.64
SE	2.47	7.13	13.02	0.06	1.59	1.56	35.14	32.32	8.72	3.06	0.37

SE = Standard error of the mean.

Table S2. Metal concentrations in groundwater, hair, fingernails, and urine of CK.

Statistical Value	Total Concentration										
	**Cr**	**Mn**	**Fe**	**Co**	**Ni**	**Cu**	**Zn**	**As**	**Mo**	**Ba**	**Pb**
**Groundwater (μg·L^{-1})**											
Min	<LOQ	154	196	<LOQ	<LOQ	<LOQ	<LOQ	402	2.25	538	<LOQ
Max	1.04	812	4962	0.95	2.94	2.02	53.42	923	6.86	1463	2.96
Mean	0.49	356	3702	0.34	0.59	0.55	10.93	686	3.43	718	0.45
Median	0.44	286	3719	0.35	0.45	0.41	6.46	700	3.26	665	0.17
SE	0.04	25.94	131.60	0.02	0.09	0.07	1.82	19.23	0.13	28.19	0.09
**Hair (mg·kg^{-1})**											
Min	0.26	2.25	31.82	0.03	0.48	6.26	131	0.65	0.08	1.06	0.70
Max	4.96	61.44	752	0.36	10.13	40.68	1208	56.75	0.68	69.19	65.21
Mean	0.90	18.17	90.62	0.08	2.31	17.73	422	9.69	0.18	17.55	6.59
Median	0.60	14.06	64.80	0.60	1.58	17.25	341	6.37	0.15	13.67	2.73
SE	0.14	2.12	14.76	0.01	0.31	0.89	36.29	1.44	0.02	1.86	1.68

Table S2. *Cont.*

Statistical Value	Total Concentration										
	**Cr**	**Mn**	**Fe**	**Co**	**Ni**	**Cu**	**Zn**	**As**	**Mo**	**Ba**	**Pb**
**Nails (mg·kg^{-1})**											
Min	0.52	1.52	109	0.04	0.50	2.84	53.30	0.77	<LOQ	1.07	0.30
Max	5.52	318	5567	1.32	48.25	91.13	393	23.24	0.32	170	27.68
Mean	1.89	31.36	1035	0.37	2.46	7.01	125	5.47	0.06	15.32	2.30
Median	1.67	19.98	782	0.31	1.34	4.57	112	3.84	0.04	10.48	1.32
SE	0.16	6.83	138.46	0.04	0.96	1.81	8.35	0.72	0.01	3.45	0.57
**Urine (ng·mg^{-1} creatinine)**											
Min	0.49	0.19	8.53	0.06	1.28	2.84	47.11	22.26	5.97	0.75	0.92
Max	21.14	272	512	3.64	70.22	55.43	872	448	198	534	16.84
Mean	2.51	7.79	65.34	0.45	11.04	12.81	328	81.07	71.98	25.51	3.02
Median	1.31	0.87	36.48	0.33	3.86	10.66	316	64.40	65.12	8.27	2.04
SE	0.52	5.46	12.72	0.07	1.68	1.26	24.80	8.64	5.73	10.90	0.44

SE = Standard error of the mean.

Table S3. Metal concentrations in groundwater, hair, fingernails, and urine of PC.

Statistical Value	Total Concentration										
	**Cr**	**Mn**	**Fe**	**Co**	**Ni**	**Cu**	**Zn**	**As**	**Mo**	**Ba**	**Pb**
	**Groundwater (μg·L^{-1})**										
Min	<LOQ	134	665	<LOQ	<LOQ	<LOQ	2.45	57.93	1.20	81.69	<LOQ
Max	1.46	2063	15057	0.89	1.59	4.15	23.33	997	5.70	2631	1.37
Mean	0.72	393	6606	0.50	0.48	0.71	6.93	587	3.67	846	0.44
Median	0.66	348	6594	0.56	0.49	0.66	6.48	650	3.19	861	0.27
SE	0.05	52.54	484.35	0.03	0.04	0.11	0.59	32.68	0.20	69.95	0.05
	**Hair (mg·kg^{-1})**										
Min	0.12	1.70	18.48	0.01	0.13	4.30	65.02	0.27	0.03	2.61	0.50
Max	14.98	156	134662	0.43	5.52	44.02	2350	22.69	0.33	233	117
Mean	0.86	48.26	2878	0.13	0.93	19.35	376	7.37	0.11	36.88	8.57
Median	0.35	46.25	161	0.11	0.64	19.47	274	6.78	0.09	24.51	3.66
SE	0.31	4.53	2689.54	0.01	0.13	1.36	50.84	0.79	0.01	5.92	2.40
	**Nails (mg·kg^{-1})**										
Min	0.54	3.28	205	0.07	0.50	2.90	64.19	1.14	0.02	3.21	0.56
Max	5.97	123	3992	1.32	41.27	30.56	202	18.48	0.13	44.72	10.03
Mean	2.11	43.00	1316	0.51	2.57	6.19	115	4.72	0.06	15.37	1.62
Median	1.85	35.87	1093	0.48	1.71	5.54	112	3.40	0.05	13.45	1.17
SE	0.17	4.58	125.56	0.05	0.80	0.59	4.01	0.49	0.01	1.32	0.21
	**Urine (ng·mg^{-1} creatinine)**										
Min	0.40	0.26	11.70	0.15	4.24	5.06	106	52.51	14.89	4.10	0.85
Max	46.02	27.68	2879	1.44	4853	109	1320	689	226	81.11	8.39
Mean	4.54	2.82	133	0.49	113	14.68	414	199	88.57	18.21	3.21
Median	2.06	1.27	44.75	0.38	10.37	12.62	371	159	78.16	13.04	2.58
SE	1.10	0.60	57.59	0.04	96.78	2.00	32.60	20.63	7.61	2.18	0.28

SE = Standard error of the mean.

Table S4. Metal concentrations in groundwater, hair, fingernail, and urine of PS.

Statistical Value	Total Concentration										
	**Cr**	**Mn**	**Fe**	**Co**	**Ni**	**Cu**	**Zn**	**As**	**Mo**	**Ba**	**Pb**
**Groundwater (μg·L^{-1})**											
Min	0.49	46.39	423	<LOQ	<LOQ	<LOQ	0.93	16.02	1.30	24.55	<LOQ
Max	3.97	897	9677	0.82	1.57	1.65	21.02	959	3.69	580	1.54
Mean	1.33	290	4479	0.27	0.82	0.49	6.54	567	2.28	222	0.37
Median	1.00	251	3988	0.16	0.77	0.35	3.90	616	2.16	150	0.17
SE	0.14	30.29	371.78	0.03	0.05	0.04	0.83	32.04	0.11	23.57	0.05
**Hair (mg·kg^{-1})**											
Min	<LOQ	2.65	24.83	0.01	0.11	2.83	36.83	0.23	0.02	<LOQ	0.35
Max	0.59	110	842	0.23	3.60	27.69	1033	12.94	0.16	31.01	17.59
Mean	0.23	25.27	133	0.06	0.66	9.39	257	4.19	0.06	9.05	3.64
Median	0.18	19.67	100	0.05	0.43	9.42	189	4.12	0.05	7.12	2.21
SE	0.02	3.27	17.50	0.01	0.09	1.46	25.31	0.36	0.01	1.02	0.57
**Nails (mg·kg^{-1})**											
Min	0.57	5.30	220	0.07	0.64	2.43	59.10	0.55	0.02	1.81	0.31
Max	6.53	113	4218	1.61	6.36	15.04	258	14.41	1.58	43.22	29.07
Mean	1.93	43.92	1434	0.49	1.93	5.92	120	4.29	0.08	13.09	2.41
Median	1.58	33.51	1059	0.41	1.63	4.99	119	3.61	0.05	10.08	1.52
SE	0.18	4.35	146.63	0.05	0.16	0.37	4.98	0.45	0.03	1.29	0.58
**Urine (ng·mg^{-1} creatinine)**											
Min	0.47	0.35	9.92	0.16	3.73	3.97	118	46.76	7.37	2.09	0.49
Max	38.20	28.16	534	2.43	38.17	51.00	2062	407	178	1402	14.39
Mean	3.36	3.42	73.90	0.51	12.34	14.81	465	103	75.78	58.08	3.40
Median	1.64	1.69	34.15	0.32	9.34	11.04	379	84.02	62.51	11.85	2.07
SE	0.82	0.67	16.30	0.07	1.27	1.46	44.43	8.97	5.66	29.08	0.48

SE = Standard error of the mean.

© 2015 by the authors; licensee MDPI, Basel, Switzerland. This article is an open access article distributed under the terms and conditions of the Creative Commons Attribution license (http://creativecommons.org/licenses/by/4.0/).
